# A case study of recurrent myocardial infarction secondary to socioeconomic challenges and nonadherence: Pre-discharge screening

**DOI:** 10.1177/2050313X231162921

**Published:** 2023-03-21

**Authors:** Revanth Kosaraju, Sami Natour, Stanley Yuan, Geoffrey Cho

**Affiliations:** 1 David Geffen School of Medicine at UCLA, Los Angeles, CA, USA; 2 Internal Medicine Residency Program, UCLA Health, Los Angeles, CA, USA; 3 Cardiovascular Research Foundation of Southern California, Beverly Hills, CA, USA

**Keywords:** STEMI, socioeconomic status, adherence, screening

## Abstract

Medication nonadherence following myocardial infarction (MI) is prevalent and increases the risk of recurrent cardiovascular events. Socioeconomic factors including medication cost, financial insecurity, and poor health literacy are associated with nonadherence. We present a patient with a history of recurrent MI who was nonadherent due to socioeconomic challenges. Our patient subsequently developed ST-elevation MI secondary to in-stent thrombosis. This case illustrates the importance of pre-discharge screening for barriers to adherence.

## Introduction

ACC/AHA guidelines recommend several medications following myocardial infarction (MI) for secondary prevention.^1^ As Kolandaivelu and colleagues note through examination of registries, nonadherence is prevalent. For instance, it is estimated that only 66% of patients used cardiovascular medications post-MI in the PREMIER registry. Other estimates are more conservative: the Ontario Database highlights that over 2 years, only 36% of patients were adherent with prescription fills post-MI for secondary prevention.^2^ Patients who are nonadherent have a greater risk of recurrent cardiovascular events and mortality.^2^ Analysis of patients in PREMIER found that those who discontinued all medications post-MI had an 11.5% risk of all-cause mortality at 1 year, compared with 2.3% in patients who were adherent to at least 1 medication.^3^ This increase in mortality was also found in patients who underwent drug-eluting stent (DES) placement and discontinued P2Y12 inhibitors.^4^ Financial barriers are commonly implicated.^5^

The objectives of this report are as follows:

To demonstrate medication affordability as a barrier to adherence.
To emphasize pre-discharge screening for barriers as an opportunity to improve outcomes.

## Case

A 72-year-old man with a history of coronary artery disease with multiple episodes of MI, heart failure with reduced ejection fraction (HFrEF), hypertension, hyperlipidemia, and a 20 pack-year history of smoking presented with chest pain.

The patient had been hospitalized three times for MI, including 2 months prior for anterior ST segment elevation MI (STEMI). In each presentation for recurrent MI, medication nonadherence was implicated as a contributor. During his most recent hospitalization for MI, he underwent placement of two current-generation DES to his mid-distal left anterior descending artery and an additional DES to a ramus intermedius. Previously placed stents were patent with otherwise non-obstructive disease. He was discharged on aspirin, atorvastatin, lisinopril, metoprolol succinate and ticagrelor.

On follow-up visit, he reported taking only aspirin and ticagrelor samples as he could not afford his other medications, and he was provided with additional ticagrelor samples. On a subsequent admission, he was changed from ticagrelor to clopidogrel due to medication affordability.

In the hours before index admission, the patient developed severe, pressure-like chest pain which radiated to both arms and was accompanied by nausea, emesis, and diaphoresis. During Emergency Medical Services evaluation, he had a blood pressure over 210/100. In the emergency department, he had signs of volume overload including jugular venous distension, elevated brain natriuretic peptide to 561 pg/mL (reference range < 100 pg/mL), and pulmonary vascular congestion on chest radiograph. He had only been taking aspirin due to cost and difficulty obtaining other medications. Electrocardiogram revealed ST elevations in II, III, aVF, V5 and V6, with an elevated troponin I to 5.3 ng/mL (reference range < 0.05 ng/mL). Emergent coronary angiography revealed thrombosis of a previously placed stent of the distal circumflex extending into the second obtuse marginal artery, which was successfully revascularized by percutaneous transluminal coronary angioplasty and serial ballooning. Stents in other vessels were patent.

Following revascularization, he was noted to be in acute decompensated heart failure and hypertensive emergency and was stabilized with a nitroglycerin drip and intravenous diuresis. While he had received a loading dose of ticagrelor, given affordability concerns, he was loaded with clopidogrel. He subsequently started medical therapy, including metoprolol succinate, atorvastatin, aspirin, lisinopril and continued clopidogrel.

Screening revealed several barriers to adherence. He noted a fixed $950 monthly income and struggled with transportation. We provided Social Work assistance with medications and Case Management assistance with obtaining Durable Medical Equipment. Home Health and transportation resources were offered, and outpatient Cardiology follow-up was arranged to optimize guideline-recommended medications for his HFrEF. Pre-discharge education was provided to help ensure medication adherence. Since his discharge, there have been no further hospital admissions.

## Discussion

Cost is a barrier to medication adherence after MI. For Medicare Part D patients, ticagrelor’s out-of-pocket cost can be up to $600 yearly.^6^ For commercially insured patients, ticagrelor or prasugrel are estimated to have over twice the yearly out-of-pocket cost of clopidogrel ($556 or $557 vs $251, respectively). Dayoub et al.^7^ have shown that differential out-of-pocket costs for P2Y12 inhibitors may contribute to differences in adherence. Unfortunately, for part D recipients with concomitant MI and HFrEF, the out-of-pocket costs of guideline-recommended medications can be over $3000 annually.^8–10^ Our patient noted that medication cost after his prior MIs played a role in his nonadherence, increasing risk for recurrent MIs.

Vouchers for copayments increase patient-reported adherence to P2Y12 agents.^11^ This coverage for copayments may increase adherence to other medications after MI.^12^ Furthermore, patients receiving full coverage had a reduced incidence of total major vascular events or revascularization.^12^ Several resources are available for patients to obtain medications more affordably. For commercially-insured patients, manufacturers provide savings cards for some brand name medications such as Brilinta.^6^ Patients with lower socioeconomic status may be eligible for Medicaid or managed Medicaid programs, though these programs often have prior authorization requirements for medications not listed on formulary. Furthermore, pharmacies such as Walmart Pharmacy^13^ have piloted monthly prescription plans with costs as low as $4 per 30-day supply, though these offers are often limited to generic medications at specific dosages. Finally, online pharmacies such as Cost Plus Drugs,^14^ while not offering newer brand-name medications such as Brilinta and Entresto, provide medications at a fraction of their cost elsewhere by negotiating with manufacturers rather than pharmacy benefit managers.

Each of our patient’s prior hospitalizations for MI afforded an opportunity for pre-discharge screening to uncover his challenges with health literacy, transportation and finances. One study estimated that only 24% of hospitals reported screening for such socioeconomic factors.^15^ Routine screening for low health literacy is associated with increased post-discharge medication adherence after MI.^16^ Following our screening, our patient was connected to programs to navigate the health system. His avoidance of hospitalizations since his discharge (over 1 year) is likely in part attributable to the screening and interventions provided during index admission.

Due to our patient’s struggles with adherence, he was frequently admitted for recurrent MI that was treated appropriately with stent placement. Ultimately, if pre-discharge screening were conducted on prior hospitalizations before the index admission to our center, interventions to address barriers revealed by such screening may have increased his medication adherence. Improved adherence then may have lowered his risk of further MI that necessitated repeat percutaneous coronary intervention and stenting, subsequently reducing his chances of developing a complication such as in-stent thrombosis which prompted index admission. We propose a multidisciplinary pre-discharge screening assessment that involves collaboration between physicians, case managers, social workers, pharmacists, nursing staff and patients. This screening can evaluate for multiple socioeconomic barriers such as the ones our patient experienced, including but not limited to financial difficulties, lack of available transportation to follow-up appointments or a pharmacy to obtain medications and limited health literacy. Then, we believe that at least some of these barriers can be systematically addressed. For example, in the case of financial barriers that make it challenging for patients to take several medications, physicians can collaborate with the other health professionals above to prescribe only the most essential medications, with a focus on finding generic equivalents or using industry-provided support to reduce costs for patients. With respect to transportation difficulties, ride-sharing or transportation vouchers are potential resources.

## Conclusion

In summary, this case illustrates the life-saving potential of pre-discharge screening to identify financial and other barriers to medication adherence. We outline some of the challenges faced by patients affected by cardiovascular disease that limit medication adherence and detail a potential interdisciplinary strategy for pre-discharge screening to improve adherence accordingly.
